# Novel Picornavirus Detected in Wild Deer: Identification, Genomic Characterisation, and Prevalence in Australia

**DOI:** 10.3390/v13122412

**Published:** 2021-12-02

**Authors:** Jose L. Huaman, Carlo Pacioni, Subir Sarker, Mark Doyle, David M. Forsyth, Anthony Pople, Teresa G. Carvalho, Karla J. Helbig

**Affiliations:** 1 Department of Physiology, Anatomy, and Microbiology, School of Life Sciences, La Trobe University, Melbourne, VIC 3086, Australia; j.huamantorres@latrobe.edu.au (J.L.H.); s.sarker@latrobe.edu.au (S.S.); t.carvalho@latrobe.edu.au (T.G.C.); 2 Department of Environment, Land, Water, and Planning, Arthur Rylah Institute for Environmental Research, Heidelberg, VIC 3084, Australia; carlo.pacioni@delwp.vic.gov.au; 3 Environmental and Conservation Sciences, School of Veterinary and Life Sciences, Murdoch University, South Street, Murdoch, WA 6150, Australia; 4 South East Local Land Services, Bega, NSW 2550, Australia; mark.doyle@lls.nsw.gov.au; 5 Vertebrate Pest Research Unit, Department of Primary Industries, Orange Agricultural Institute, Orange, NSW 2800, Australia; dave.forsyth@dpi.nsw.gov.au; 6 Department of Agriculture and Fisheries, Invasive Plants & Animals Research, Biosecurity Queensland, Ecosciences Precinct, Brisbane, QLD 4102, Australia; tony.pople@daf.qld.gov.au

**Keywords:** *Bopivirus*, complete genome, viral metagenomics, viral isolation, phylogenetics, deer

## Abstract

The use of high-throughput sequencing has facilitated virus discovery in wild animals and helped determine their potential threat to humans and other animals. We report the complete genome sequence of a novel picornavirus identified by next-generation sequencing in faeces from Australian fallow deer. Genomic analysis revealed that this virus possesses a typical picornavirus-like genomic organisation of 7554 nt with a single open reading frame (ORF) encoding a polyprotein of 2225 amino acids. Based on the amino acid identity comparison and phylogenetic analysis of the P1, 2C, 3CD, and VP1 regions, this novel picornavirus was closely related to but distinct from known bopiviruses detected to date. This finding suggests that deer/bopivirus could belong to a novel species within the genus *Bopivirus,* tentatively designated as “*Bopivirus* C”. Epidemiological investigation of 91 deer (71 fallow, 14 sambar and 6 red deer) and 23 cattle faecal samples showed that six fallow deer and one red deer (overall prevalence 7.7%, 95% confidence interval [CI] 3.8–15.0%) tested positive, but deer/bopivirus was undetectable in sambar deer and cattle. In addition, phylogenetic and sequence analyses indicate that the same genotype is circulating in south-eastern Australia. To our knowledge, this study reports for the first time a deer-origin bopivirus and the presence of a member of genus *Bopivirus* in Australia. Further epidemiological and molecular studies are needed to investigate the geographic distribution and pathogenic potential of this novel *Bopivirus* species in other domestic and wild animal species.

## 1. Introduction

Wild animals are widely described as natural reservoir hosts for various pathogens transmitted to humans, domestic, and other wild animals. Moreover, the frequency of emerging and re-emerging infectious disease outbreaks in wildlife has increased during recent decades [1], raising new questions about disease pathogenesis and epidemiology. During the last two decades, the use of high-throughput DNA sequencing technologies has allowed the identification of novel, highly divergent viral species and those that cannot be detected by conventional culture and sequence-dependent methods [2]. Thus, it has dramatically increased viral discovery from various environmental and animal samples, including novel picornaviruses [3]. Picornaviruses are ubiquitous, small, non-enveloped positive-sense RNA viruses infecting mainly mammals and birds [3,4]. In the last year, novel picornaviruses have been reported in Magellanic penguins [5], Cherry Valley ducks [6], rats (*Rattus norvegicus* and *Rattus tanezumi*) [7], wild vervet monkeys (*Chlorocebus pygerythrus*) [8], and livestock (sheep and goat) [9].

Several viral pathogens of farm ruminants have been detected in wild cervids globally, including several of significant agricultural relevance such as *Pestivirus* [10,11]. The six non-native deer species in Australia have expanded considerably in recent years; however, there is little information regarding which pathogens are present and whether these pathogens pose biosecurity threats to humans, wildlife, livestock, or other domesticated animals. Addressing this knowledge gap is essential for anticipating how viruses might be transmitted to other animals and controlling diseases. Therefore, our team has been investigating the infection status of wild deer in Australia [11,12,13].

This research aimed to characterise viruses in deer faecal samples through metagenomics and determine the diversity and prevalence of any novel detected viruses. Here, we report the detection, genome characterisation, and isolation of a novel picornavirus in Australian wild deer that was found to be closely related to but distinct from bopiviruses detected to date. Further, an RT-PCR-based epidemiological investigation was conducted using faecal samples of wild deer and cattle collected in south-eastern Australia. The family *Picornaviridae* is very diverse, having numerous genera and genotypes. Multiple picornaviruses have been detected previously among deer species, including *Kobivirus* and Bovine *Enterovirus* in roe deer (*Capreolus capreolus*) [14] and white-tailed deer (*Odocoileus virginianus*) [15], respectively; however, little is known about their pathogenicity or distribution. To date, genus *Bopivirus* contains a single species *Bopivirus* A, detected in cattle; however, a novel proposed species called “*Bopivirus* B” was recently described in sheep and goats from Hungary [3,9]. Based on these findings, it is suggested that this novel picornavirus belongs to a novel species in the genus *Bopivirus*, tentatively designed as “*Bopivirus C*”.

## 2. Materials and Methods

### 2.1. Sample Collection and Preparation

**Figure 1:**
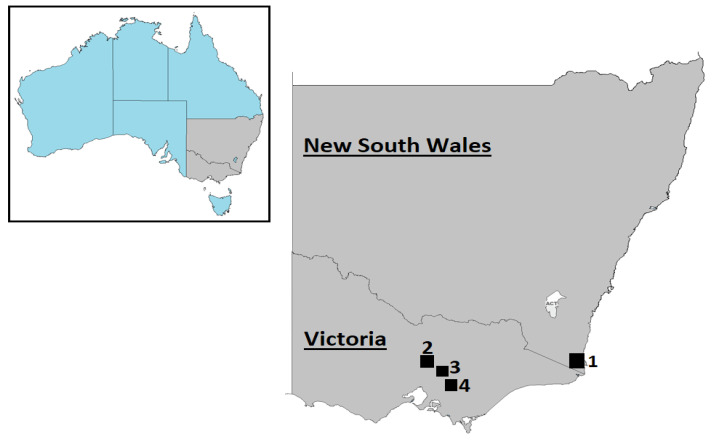
The geographic location of deer (1–4) and cattle (1, 2, and 4) sampling in south-eastern Australia. (1) Kiah, (2) Outer Melbourne, (3) Yellingbo, and (4) Bunyip. ©d-maps.com (accessed on 2 August 2021).

This study utilised previously collected samples to investigate Australian wild deer infection status [11,12,13]. Briefly, opportunistic sampling during field necropsies was carried out on deer culled for other reasons in New South Wales and Victoria (Figure 1) from August 2019 to June 2021 with the assistance of recreational and professional hunters. Ninety-one faecal samples were collected from wild deer comprising 71 fallow deer (*Dama dama*), 14 sambar deer (*Rusa unicolor*), and six red deer (*Cervus elaphus*). All samples were collected from the large intestine and individually placed in sterile plastic containers. Additionally, 23 opportunistic cattle faecal samples collected for other purposes (i.e., clinical purposes) from farms within 20 kilometres of deer sampling areas were included in the present study. All sample containers were immediately refrigerated after collection, then transported to the Laboratory of Virology within the Department of Physiology, Anatomy, and Microbiology at La Trobe University. 0.1 g of each specimen was placed in RNase-free tubes with 1 mL of phosphate-buffered saline (PBS; 10% *w*/*v*) and kept frozen at −80 °C until further use. After homogenisation, faecal suspensions were centrifuged at 4000× *g* for 15 min at 4 °C to collect the supernatants. To avoid contamination of our results, we employed a laminar flow cabinet equipped with a UV lamp for aliquoting and preparing samples.

### 2.2. Viral Metagenomic Analysis

Twelve deer faecal samples across the regions (9 fallow deer and three sambar deer) were randomly selected and sequenced through next-generation sequencing. Each supernatant was filtered through 0.8 μm polyethersulfone (PES) (Sartorius cat#: VK01P042) spin filters at 17,000× g for 1 min. Filtrates were nuclease treated using benzonase and micrococcal nuclease (37 °C for 2 h), followed by nucleic acid extraction using the QIAamp^®^ Viral RNA mini kit (Qiagen, Valencia, CA, USA) as previously described [13]. cDNA synthesis and amplification were performed using the Whole Transcriptome Amplification Kit (WTA2, Sigma-Aldrich, Darmstadt, Germany). Sequencing libraries were constructed using the Nextera XT Flexi DNA Library kit (Illumina, San Diego, CA, USA) according to the manufacturer’s instructions. Libraries were then pooled and sequenced with the Illumina NovaSeq platform using a 2 × 150 PE high-output flow cell at the Australian Genome Research Facility, Melbourne, Australia.

### 2.3. Bioinformatic Analysis

Bioinformatics analysis was conducted as described previously [13]. Briefly, Illumina reads were demultiplexed and trimmed using Trim_Galore v0.4.5 with the minimum sequence length set to 50 bp and Phred Quality of 25. Good quality reads were used to produce de novo assemblies with SPAdes v3.10.1. The resulting contigs were compared against the nonredundant nucleotide database on GenBank using BLASTn (e-value threshold of 1 × 10^−5^). Viral contigs of interest (>500 nucleotides and query cover >50%) were imported in Geneious software (Biomatters Ltd., Auckland, New Zealand, version 11.1.4). Reads were subsequently mapped back to viral contigs using the Burrows–Wheeler Aligner BWA-MEM (v 0.7.17).

### 2.4. Genomic Characterisation

Gene prediction was performed using ORFfinder (https://www.ncbi.nlm.nih.gov/orffinder/ accessed on 3 September 2021). Protein domains were predicted using InterProScan [16] and Pfam conserved domain search [17]. Internal ribosomal entry site (IRES) was assessed using IRESPred [18] and Rfam sequence search tool [19]. Possible proteolytic cleavage sites of the study viruses were predicted based on the individual amino acid alignments with other described bopiviruses [9]. Relevant motifs in the polyprotein were identified using NCBI conserved domain search (https://www.ncbi.nlm.nih.gov/Structure/cdd/wrpsb.cgi accessed on 3 September 2021).

### 2.5. RT-PCR Detection of Deer/Bopivirus

Standard precautions to avoid product contamination were taken for all PCR assays, including filter pipette tips and physically separated rooms for PCR setup and analysis. RNA was extracted from faecal samples using QIAamp^®^ Viral RNA Mini Kit (Qiagen, Valencia, CA, USA), then cDNA synthesis using Tetro cDNA Synthesis Kit (Bioline, London, UK) with random hexamers. RNA-negative samples (nuclease-free water) were also included for each extraction round and negative controls in every PCR run. Primers CTGRGCAAGTTCACCAACAA and GTCCATGACAGGGTGAATCA [9] were used to detect the 3D gene by amplifying a PCR product of 627 bp. In addition, a 432bp fragment of the VP1 gene was obtained by custom primers TGTTGACGCGTCTGACATGA and ATCCCTGGTGGGGAGTAGAC, corresponding to position 2515 to 2534 and 2927 to 2946 of deer/VIC82-2020/AUS, respectively. PCR amplification was performed in a 25 μL reaction mixture containing 1× Green GoTaq Flexi buffer, 2 mM of MgCl2, 10 mM of dNTPs, 0.2 μM of both forward and reverse primers, 0.625 units of GoTaq G2 DNA polymerase (Promega, Madison, WI, USA), and 1 μL of cDNA. PCR conditions were 95 °C for 2 min, 40 cycles of 95 °C for 30 s, 50 °C (3D) or 56 °C (VP1) for 30 s, 72 °C for 45 s, with a final extension at 72 °C for 5 min. PCR-positive products were purified using the Wizard^®^ SV Gel and PCR Clean-Up kit (Promega, Madison, WI, USA) according to the manufacturer’s instructions. Sanger sequencing was performed at the Australian Genome Research Facility, Melbourne, Australia. Viral prevalence was calculated from the proportion of positive results and presented with 95% confidence interval (CI), calculated using the Wilson score interval (www.epitools.ausvet.com.au accessed on 28 November 2021).

### 2.6. Phylogenetic Analysis

Nucleotide sequences were edited and translated using Geneious software (Biomatters Ltd., Auckland, New Zealand, version 11.1.4). Amino acid sequences of deer/bopiviruses identified in the present study were compared to the corresponding sequences of other bopiviruses (MW298057, MW298058, MW298059, KM589358) and representative strains of picornaviruses (KX260139, FJ175661, MH976711, AY421763, LC055960). Multiple sequence alignments were performed using Clustal X [20]. The best-fitting substitution model was determined based on the lowest BIC scores in MEGA 7 [21]. Phylogenetic trees were constructed with this software, using the maximum-likelihood method. Statistical support for the trees was evaluated by bootstrapping based on 1000 repetitions.

### 2.7. Virus Inoculation in Cell Cultures

Mardin–Darby bovine kidney (*Bos taurus*, MDBK) and Vero (*Cercopithecus aethiops*) cell lines were used to culture viruses at 37 °C in a 5% CO_2_ humidified atmosphere in either Eagle’s Minimum Essential Medium (EMEM) supplemented with 5% fetal bovine serum (FBS) or in Dulbecco’s Modified Eagle Medium (DMEM) supplemented with 10% FBS, respectively. Faecal suspensions (~20 *v*/*v*% in 0.1 M PBS) used for inoculations were first centrifuged (10,000 rpm for 10 min at 4 °C) then the supernatants were passed through 0.45 µm sterile membrane syringe filters (Millipore, Bedford, MA, USA). Then, 1 mL of 1:10 diluted filtrate was inoculated into T25 cm^2^ cell culture flasks with 80–90% cell confluency for 1 h at 37 °C, followed by the addition of fresh medium. Negative controls without faecal specimens were also used. After 24 h post-infection, the flask supernatant was removed, and the monolayer was rinsed once using EMEM, prior to incubation in complete medium. Cultures were inspected daily by inverted microscopy for cytopathic effect (CPE). After 14 days post-infection or once the cytopathic effect was observed, the cells were frozen and thawed once, and the culture lysates were collected for RNA extraction. The presence of deer/bopivirus RNA was verified using RT-PCR as described above.

### 2.8. Data Availability

The nucleotide sequences of deer/bopivirus detected in this study were submitted to GenBank under accession number MZ436972 (complete genome—deer/VIC82-2020/AUS), OK143288-OK143294 (partial VP1 region), and OK143295-OK143299 (partial 3D region).

## 3. Results

### 3.1. High-Throughput Sequencing and Assembly

Illumina sequencing of twelve deer faecal samples generated a total of 269,043,972 paired-end (PE) reads (range 11,918,979–37,909,513 PE reads), representing a total of 81.26 gigabases of sequence data. After trimming, 98.7% of the reads (265.5 million, range 11,729,223–37,488,996 PE reads) were retained (Table S1). Then, we de novo assembled an average of 10,228 contigs, of which 4% comprised viral contigs. From the total number of viral contigs, phages and single-stranded DNA (CRESS DNA) viruses made up at least 95% (data not shown). Further analysis of eukaryotic viral contigs revealed matching with genus *Bopivirus* (family *Picornaviridae*) in two deer samples with lengths > 500 bp. Additionally, a third sample showed a contig belonging to the same viral genus; however, it was <500 bp in length. These three contigs displayed the closest relatedness with members in the genus *Bopivirus* previously identified in cattle from the USA (KM589358) and Hungary (MW298059) (Table 1).

### 3.2. Genomic and Phylogenetic Analysis of Bopivirus-Like Sequence

**Figure 2:**
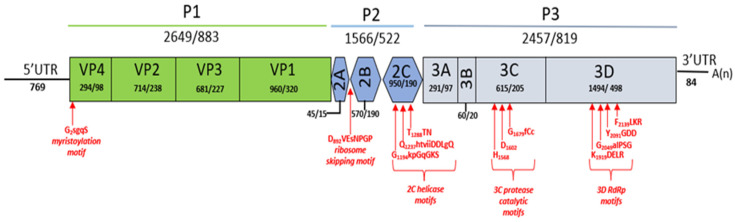
Genome organisation of deer/bopivirus described in this study. The gene boxes corresponding to the P1 (viral capsid proteins), P2, and P3 (non-structural proteins) are highlighted differently. The nucleotide and amino acid lengths of the corresponding genomic regions are shown in each gene box. A red arrow indicates relevant protein motifs and their position within the polyprotein.

By de novo assembly, a 7554 bp *Bopivirus* genome (deer/VIC82-2020/AUS, MZ436972) was successfully obtained from fallow deer, herein called deer/bopivirus, for descriptive purposes in the present study. This sequence included a 5′ untranslated region (UTR) of 769 nt, one complete ORF of 6675 nt encoding a polyprotein of 2225 amino acids, a 3′ UTR of 84 nt, and a polyA-tail (Figure 2). The virus genome had a GC content of 51.3%, excluding the poly(A) tail. Using InterProScan domain prediction, we have predicted three precursors, P1, P2, and P3 within the deer/bopivirus polyprotein. Further, we were able to identify all putative cleavage sites of the polyprotein using alignments with other known viruses of the genus *Bopivirus* as per Laszlo et al. [9]. Most of the cleavage sites are conserved among all bopiviruses; however, differences were found between VP3/VP1, VP1/2A, 3A/3B, and 3C and 3D (Table S2). Based on this result, the polyprotein of deer/bopivirus consists of four structural proteins and seven non-structural proteins (Figure 2).

As reported for bopiviruses, the start codon of the polyprotein contains a Kozak consensus sequence (ttCACaA_770_TGG, start codon is underlined, conserved nucleotides are capitalised) [9]. Moreover, the N-terminal end of VP4 is myristoylated, and the single, short (15 aa-long) 2A peptide contains the “ribosome skipping” site of DxExNPGP (x = variable aa, conserved aa are capitalised), indicating that deer/bopivirus also belongs to the aphthovirus-like 2A-type [9,22,23]. Using IRESPred, a potential IRES was identified in deer/bopivirus, and Rfam search revealed that it belongs to type 2 with homology to Aphthovirus IRES. The 5′ UTR and 3′ UTR of deer/bopivirus shared 89% and 82.5% nt identity to *Bopivirus* bovine/TV-9682/2019-Hun (MW298059), respectively. In addition, no sequence repeats or conserved motifs were detected in the 3′ UTR. The polyprotein of deer/bopivirus shows 78–79% nt and 79% aa sequence identity to bopiviruses detected in cattle, which are the closest match identifiable with BLAST searches. However, lower aa identities were found between deer/bopivirus and bopiviruses detected in sheep and goats (Table 2). Pairwise alignment of the amino acid sequences of deer/bopivirus and other reported bopiviruses shows >33% identities for capsid region P1 and non-structural proteins 2C and 3CD (Table 2). This criterion is currently used by the International Committee on Taxonomy of Viruses (ICTV) to classify members of the same picornaviral genus.

**Figure 3:**
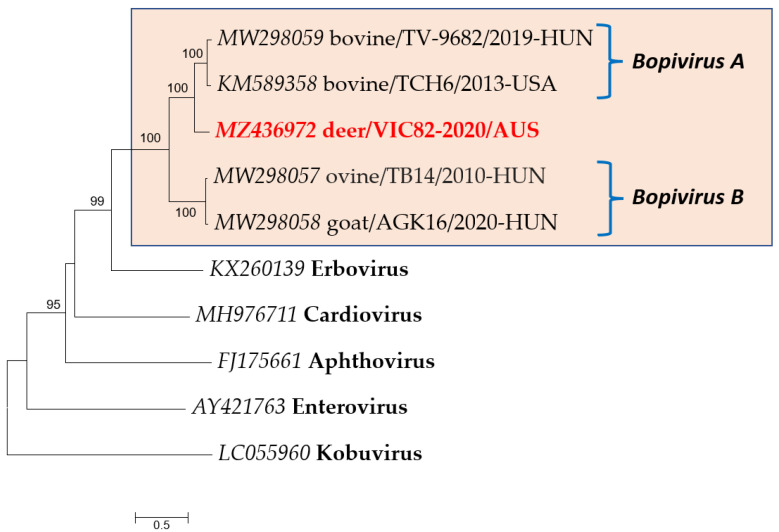
Phylogenetic analysis of deer/bopivirus (in red) and representative member of family *Picornaviridae* based on complete polyprotein aa sequences. A highlighted box denotes members of the genus *Bopivirus*. The tree was generated by the maximum-likelihood method based on LG + G substitution model with 1000 bootstrap replicates, and the statistics values > 70% are displayed above the tree branches. The scale bar indicates amino acid substitutions per site.

The phylogenetic tree based on the complete polyprotein aa sequences of deer/VIC82-2020/AUS and representative picornaviruses shows that deer/bopivirus was closely related to but distinct from bopiviruses detected to date (Figure 3). Bopiviruses detected in cattle, sheep, goat, and deer grouped in a monophyletic clade with high bootstrap support; however, deer/bopivirus forms a distinct cluster. Similar tree topology was found based on P1, 2C, 3CD, and VP1 aa sequences supported by high bootstrap values in all three phylogenetic trees (Figure S1). Together with pairwise comparison, these findings suggest deer/bopivirus could belong to a novel species in this genus *Bopivirus*.

### 3.3. Prevalence of Deer/Bopivirus

Raw data of the twelve libraries assessed by next-generation sequencing were mapped against the full-length genome of deer/bopivirus to investigate the abundance of deer/bopivirus. Eight samples showed less than 50 reads mapped with this novel picornavirus, and no mapped reads were found in 3 libraries (Table S1). Further, faeces from 91 deer and 23 cattle were screened by RT-PCR targeting VP1 and 3D genes. Overall, 7 deer (7.7%, 95% confidence interval [CI] 3.8–15.0%) were positive for deer/bopivirus RNA. PCR amplification for both genes was obtained in five samples, while the remaining two samples were positive only for the VP1 gene. None of the analysed sambar deer and cattle samples were found to be RT-PCR positive (Table 3).

**Figure 4:**
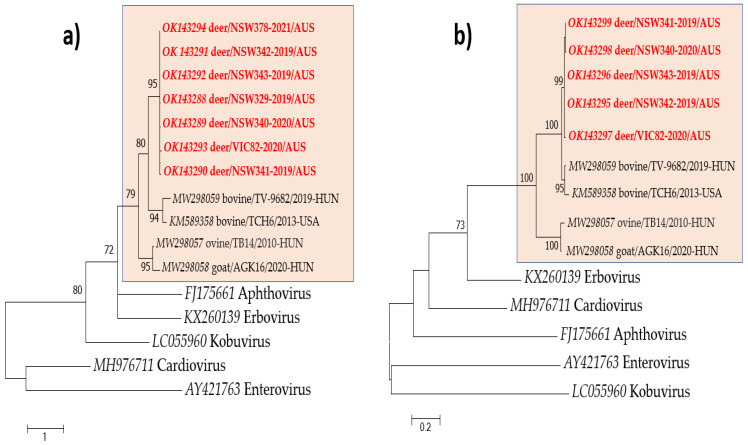
Phylogenetic analyses of deer/bopivirus (in red) and representative members of family *Picornaviridae* based on partial VP1 (**a**) and 3D (**b**) aa sequences. A highlighted box denotes members of the genus *Bopivirus*. The trees were generated by the maximum-likelihood method based on JTT + G (**a**) and LG + G (**b**) substitution model with 1000 bootstrap replicates, and the statistics values > 70% are displayed above the tree branches. The scale bar indicates amino acid substitutions per site.

Nucleotide sequences were obtained from the seven VP1 (385 nt/127 aa) and five 3D (587 nt/195 aa) PCR-positive products. Based on the results of pairwise sequence comparisons and phylogenetic analysis of partial VP1 and 3D, deer/bopivirus sequences formed a monophyletic clade (Figure 4), with sequences sharing 95.3–100% (VP1) and 98.5–100% (3D) aa identities. Moreover, bovine, ovine, and caprine bopiviruses formed sister clades to the novel deer/bopivirus.

### 3.4. Viral Culture

Two deer samples positive for deer/bopivirus RNA were assessed for virus isolation. No cytopathic effect was observed in any samples and cell lines (Figures S2 and S3). Moreover, RT-PCR resulted negative in Vero cell lysate collected at the end of culture assessment (day 14). Conversely, MDBK cell lysate revealed the presence of deer/bopivirus RNA by RT-PCR in all samples assessed, indicating that this novel virus may be culturable.

## 4. Discussion

In recent years, the use of high-throughput sequencing has facilitated virus discovery due to the potential to detect infectious agents having an unknown genetic background [2,3]. The present study reports a novel virus’s detection, genomic characterisation, and isolation in faecal samples of wild deer sampled in south-eastern Australia. Genomic characterisation showed that this virus has the same genome structure as those of typical picornaviruses, displaying the presence of a single large open reading frame flanked by UTRs. According to the current classification criteria of the International Committee on Taxonomy of Viruses (ICTV) [3,4], members of the same picornaviral genus should share >33% amino acid identity for the capsid region P1 and >36% amino acid identity for non-structural proteins 2C and 3CD. This novel picornavirus represents a new member of the genus *Bopivirus*, sharing >55% aa identities in all structural and non-structural protein regions. Furthermore, phylogenetic analysis based on polyprotein, P1, 2C, 3CD, VP1 aa sequences shows this deer-origin picornavirus is phylogenetically distinct and divergent from other bopiviruses detected to date, suggesting that deer/bopivirus could belong to a novel species within the genus *Bopivirus,* proposed as “*Bopivirus* C”.

Detailed genomic analysis showed the presence of bopivirus conserved motifs (e.g., protein cleavage sites, lack of a leader protein, P2, and P3 functional domains), with minor variance. Conserved characteristic motifs are the basis of the function and structure of picornaviruses, and some significant functional motifs were recognised in the polyprotein of deer/bopivirus. The first capsid protein (VP4) displayed the conserved sequence GXXX(S/T) for myristoylation, which plays an essential role in virion morphogenesis by covalent linkage of myristic acid to an N-terminal glycine residue [24]. Conserved sequence motifs in the 2C helicase (Walker A motif: GxxGxGKS), 3C proteinase (GxCGx10-15GxH), and 3D polymerase (KDE, DxxxxD, PSG, YGDD, FLKR) were also identified [4]. For picornaviruses, the 2A proteins were grouped into four distinct basic types based on aa sequence and/or function, including enterovirus-like 2A (trypsin-like protease), aphthovirus-like 2A containing an NPGP motif, parechovirus-like 2A harboring H-box/NC motifs, and 2A unrelated to any known proteins [25]. According to the above guideline and similarity with other bopiviruses, the 2A protein of deer/bopivirus belongs to aphthovirus-like 2A.

Phylogenetic analysis of the VP1 coding region indicates that deer/bopivirus and bopiviruses formed a cluster with high bootstrap support, even though VP1 is the most diverse capsid protein of picornaviruses [26,27]. Moreover, deer/bopivirus is most related to bopiviruses detected in cattle. They both contain a type II IRES with high nucleotide sequence similarity in the core domains H-I-J-K-L (*data not shown*). Although their host species are different, this suggests that they may have a common ancestor. As RNA viruses have a remarkable potential to evolve due to high mutation and recombination rates, it is plausible that deer/bopivirus may be derived from *Bopivirus* A and have evolved independently in deer [28].

For this study, 91 wild deer from different locations throughout south-eastern Australia were screened for the presence of deer/bopivirus RNA, with almost 8% of these animals testing positive. In addition, five out of the seven positive samples showed amplification for both target regions (VP1 and 3D). The failure to obtain an amplicon using 3D primers in two samples may be influenced by (i) the incomplete nucleotide match between the primer and the target region, as we employed generic primers designed for bopiviruses in cattle, sheep, and goats, (ii) a different limit of detection between the two primer sets, and/or, (iii) a low deer/bopivirus RNA concentration, which could result in a disadvantageous competition during RT-PCR. Phylogenetic and sequence analyses indicated that the same deer/bopivirus strain is circulating in New South Wales and Victoria. Deer/bopivirus RNA was not detected in the investigated cattle faecal samples using VP1 primers designed to detect deer/bopivirus. Negative results were also obtained using a generic bopivirus screening RT-PCR reaction (3D region) previously employed by Laszlo et al. [9] in livestock samples. This fact could indicate the absence of bopiviruses in the tested samples; however, we cannot rule out their circulation in Australian cattle due to the low sample numbers available for testing in our study.

The family *Picornaviridae* contains clinically relevant human and animal pathogens and is associated with a series of diseases in the central nervous system, respiratory tract, heart, liver, pancreas, skin, and eye [4]. All positive samples were collected from apparently healthy deer at the sampling time, suggesting that deer could be a reservoir for this novel virus. However, the presence of a virus in healthy animals does not rule out its pathogenic potential. Picornaviruses have previously been detected in both symptomatic and asymptomatic animals, with kobuviruses being isolated from a wide range of hosts, including human, wild, and domestic animals with and without clinical signs worldwide [29,30]. Additionally, caprine kobuvirus has been detected in both healthy and diarrheic goats in Italy, with a higher percentage observed in diarrheic goats [31]. The pathogenicity of deer/bopivirus in deer and other animals remains undetermined. Additionally, it is unclear if the shedding of this virus in asymptomatic deer represents a mechanism of virus persistence in the animal population.

In addition to molecular characterisation, viral isolation was performed in two separate cell lines. Neither cytopathic effect nor the expected RT-PCR products were observed in Vero cells, indicating that this cell line is unsuitable for deer/bopivirus replication. On the other hand, deer/bopivirus RNA was obtained after 14 days of incubation (end of viral culture assessment) but without cytopathology. In the absence of demonstration of an increasing viral titre via real-time PCR over two time points, we cannot conclusively state that deer/bopivirus is able to replicate in cell culture; however, this result indicates that MDBK cells of bovine origin may be suitable for deer/bopivirus growth. Nevertheless, further work is required to determine this. Although previous attempts to culture bopiviruses in MDBK cells were unsuccessful [9], incubation time may influence the failure or success of virus replication, with a 14 day incubation utilised in the present study compared with four days employed by Laszlo et al. [9].

## 5. Conclusions

We report here the first description and isolation of a novel bopivirus identified in Australian deer, expanding the host range of this viral genus. Moreover, genomic, and phylogenetic analyses indicate that this bopivirus belongs to a new species, proposed as “*Bopivirus* C”. Further epidemiological and molecular studies are required to investigate the incidence, diversity, geographic distribution, pathogenicity, and clinical importance of this novel picornavirus in deer and other domestic and wild animal species.

## Figures and Tables

**Table 1:** Percentage identities of the assembled contigs for the *Picornaviridae* family.

Sample ID	Sampling Location	Deer Species	Contig Length (nt)	Subject Cover	Best Hit (AC Number)	Perc. Identity
NSW301	Kiah-NSW	Fallow deer ^a^	332	95%	*Bopivirus* A isolate TCH6 (KM589358)	86%
				95%	*Bopivirus* sp. strain bovine/TV-9682/2019-HUN(MW298059)	82%
NSW341	Kiah-NSW	Fallow deer ^a^	644	63%	*Bopivirus* A isolate TCH6 (KM589358)	86%
				63%	*Bopivirus* sp. strain bovine/TV-9682/2019-HUN(MW298059)	86%
VIC82	Victoria	Fallow deer ^a^	7554	55%	*Bopivirus* A isolate TCH6 (KM589358)	79%
				61%	*Bopivirus* sp. strain bovine/TV-9682/2019-HUN(MW298059)	78%

^a^ *Dama dama*, NSW: New South Wales.

**Table 2:** Comparison of genomic features of deer/VIC82-2020/AUS (MZ436972) and described bopiviruses.

Virus	Accession Number	Host	Genomic Features		Pairwise Amino Acid Identity (%)				
			Size (nt)	GC Content	Polyprotein	P1	2C	3C	3D
bovine/TCH6/2013-USA	KM589358	Cattle	7018	50.4%	79	66.7	91.2	90.7	88.5
bovine/TV-9682/2019-HUN	MW298059	Cattle	7571	50.2%	78.7	66.1	91.2	90.2	89.7
ovine/TB14/2010-HUN	MW298057	Sheep	7385	54.9%	58.7	57.2	62.5	66.8	66.9
goat/AGK16/2020-HUN	MW298058	Goat	7426	55.1%	58.2	55.7	62.1	66.8	67.3

**Table 3:** Prevalence of deer/bopivirus in deer and cattle from south-eastern Australia.

Location	Fallow Deer		Sambar Deer		Red Deer		Cattle	
	N	*n* (%, CI)	N	*n* (%, CI)	N	*n* (%, CI)	N	*n* (%, CI)
New South Wales	59	5 (8.5, 3.7–18.4)	3	0 (0–56.2)	6	1 (16.7, 3.0–56.4)	8	0 (0–32.4)
Victoria	12	1 (8.3, 1.5–35.4)	11	0 (0–25.9)	0	0	15	0 (0–20.4)
Total	71	6 (8.5, 3.9–17.2)	14	0 (0–21.5)	6	1 (16.7, 3.0–56.4)	23	0 (0–14.3)

N: samples tested, *n*: positive samples, and CI: 95% confidence interval.

## Data Availability

All data can be obtained from the authors on request.

## Supplementary Materials

The following are available online at https://www.mdpi.com/article/10.3390/v13122412/s1, Figure S1. Phylogenetic analysis of deer/bopivirus (in red) based on P1, 2C, 3CD, and VP1 aa sequences. The tree was generated by the maximum-likelihood method based on LG + G substitution model with 1000 bootstrap replicates, and the statistics values > 70% are displayed above the tree branches. The scale bar indicates amino acid substitutions per site. Figure S2. Viral culture on MDBK cells. (a) not infected cells (negative control). (b) inoculated cells after 14 days of incubation. Light microscope (10X). Figure S3. Viral culture on Vero cells. (a) not infected cells (negative control). (b) inoculated cells after 14 days of incubation. Light microscope (10X). Table S1. Results of mapping reads of the twelve samples against the novel picornavirus described in the present study. Table S2. Predicted cleavage sites determined by multiple alignments with other *Bopivirus* sequences.
