# Supplementary material for: Novel Picornavirus Detected in Wild Deer: Identification, Genomic Characterisation, and Prevalence in Australia
Source: Viruses. 2021 Dec 2;13(12):2412. doi: 10.3390/v13122412 (PMC8706930; doi:10.3390/v13122412)
Supplement: Supplementary file 1 [file viruses-13-02412-s001.zip › viruses-1472950-supplementary.pdf]

Supplemental Materials

Novel Picornavirus Detected in Wild Deer: Identification, Characterisation, and Prevalence in Australia

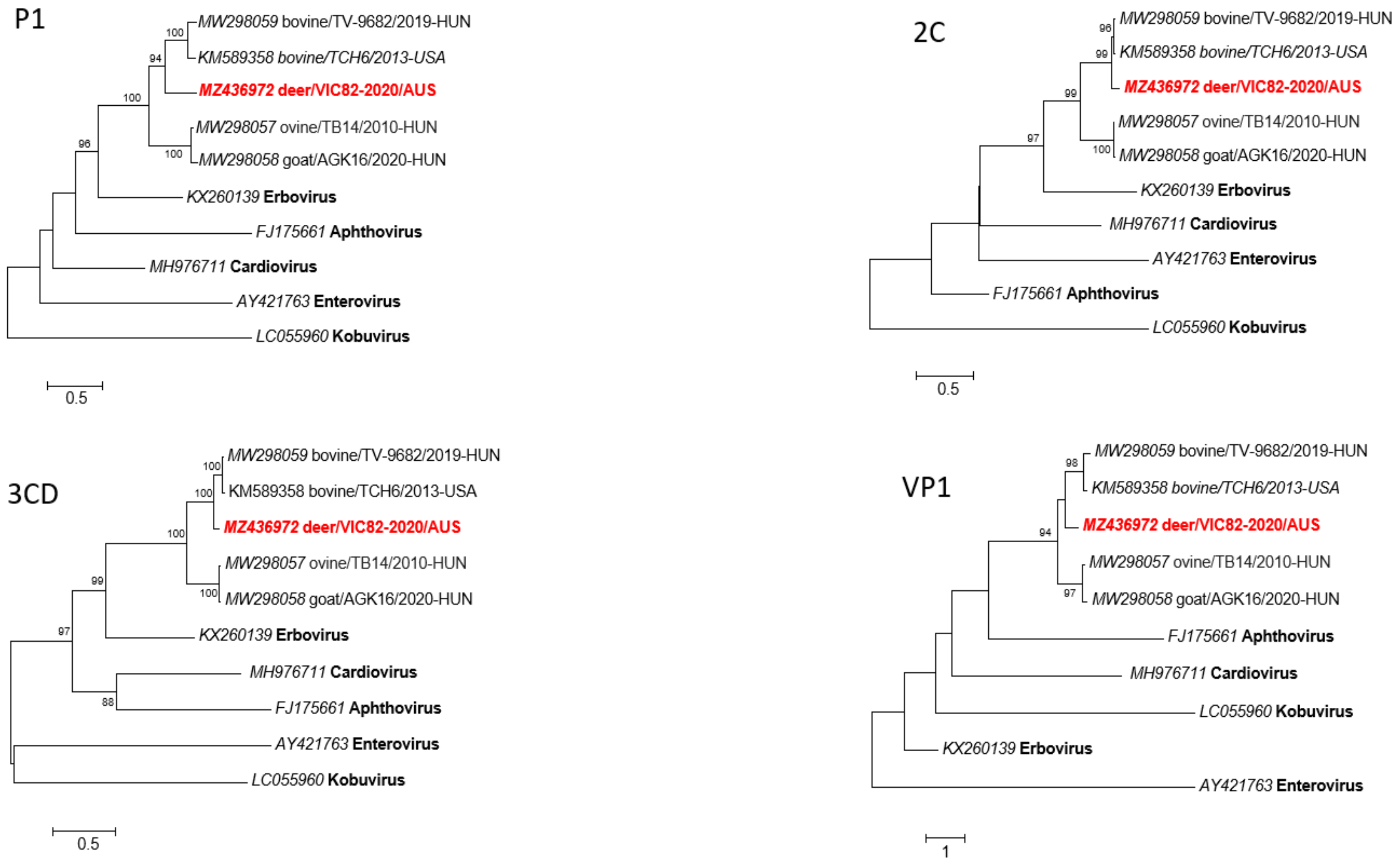

Figure S1. Phylogenetic analysis of deer/bopivirus (in red) based on P1, 2C, 3CD, and VP1 aa sequences. The tree was generated by the maximum-likelihood method based on LG + G substitution model with 1000 bootstrap replicates, and the statistics values > 70% are displayed above the tree branches. The scale bar indicates amino acid substitutions per site.

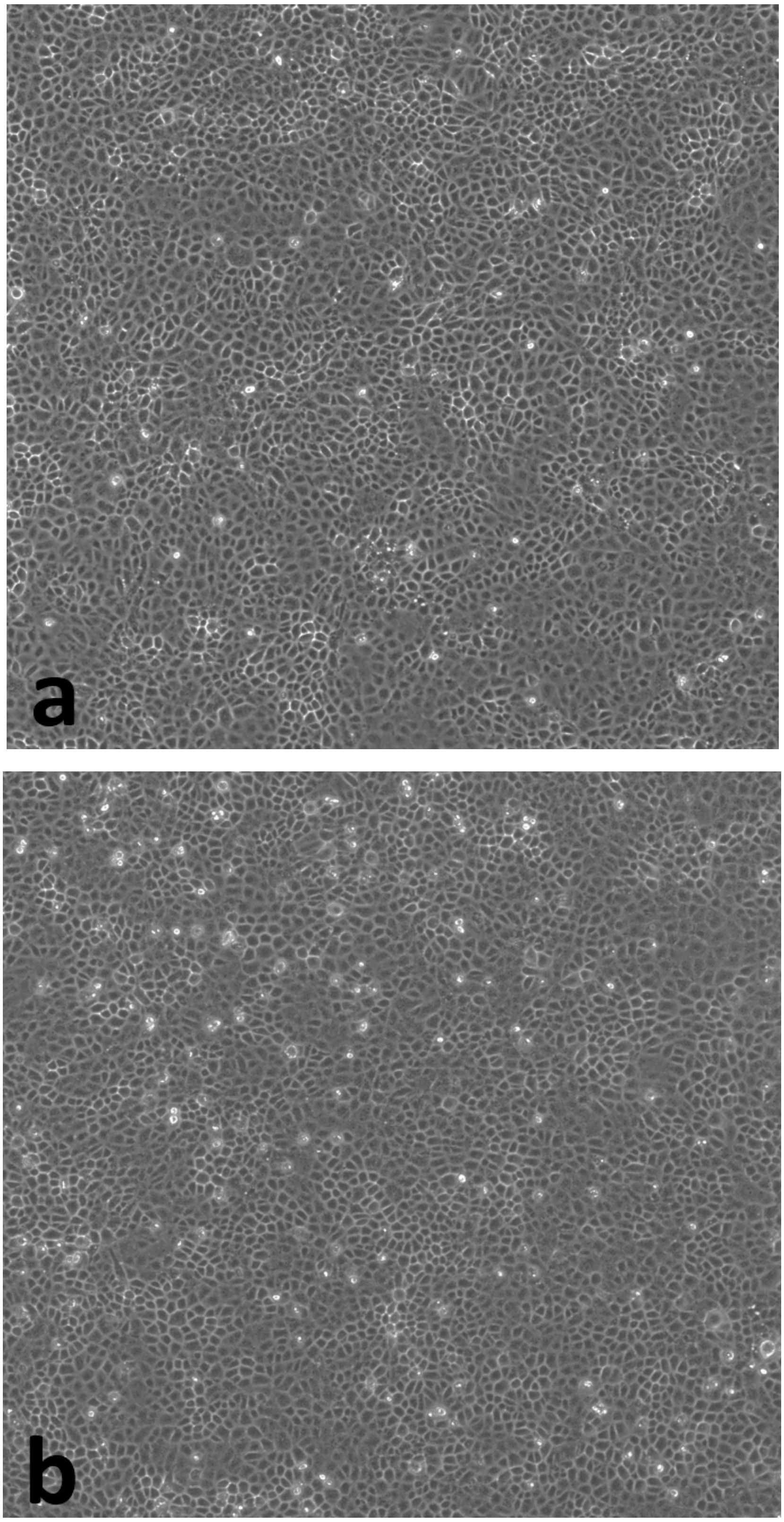

Figure S2. Viral culture on MDBK cells. a) not infected cells (negative control). b) inoculated cells after 14 days of incubation. Light microscope (10X).

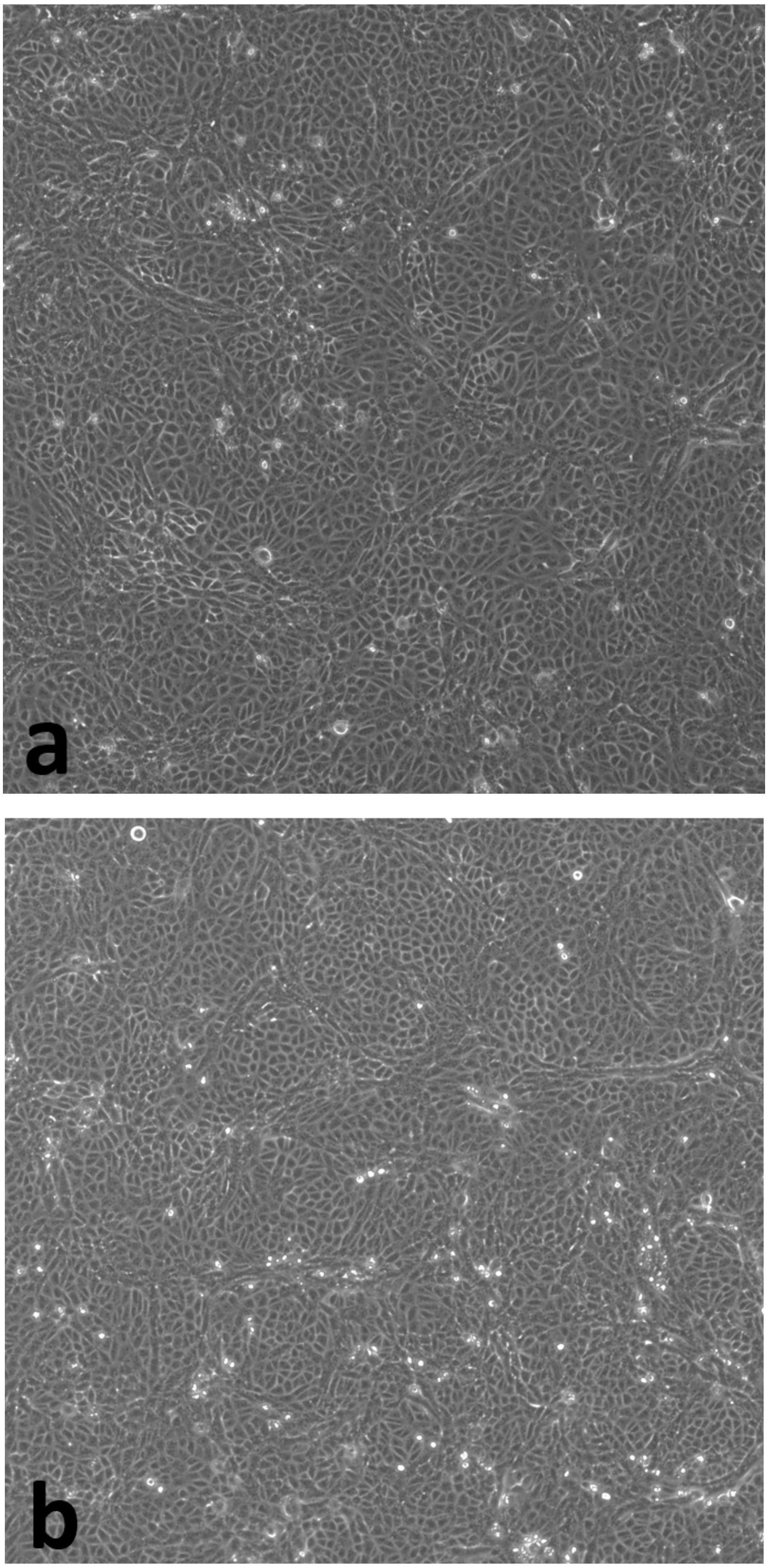

Figure S3. Viral culture on Vero cells. a) not infected cells (negative control). b) inoculated cells after 14 days of incubation. Light microscope (10X).

Table S1: Results of mapping reads of the twelve samples against the novel picornavirus described in the present study

Sample ID	Deer species	Total number of PE reads	Number of trimmed PE reads	Number of reads mapped deer/bopivirus
NSW301	fallow	25,407,009	25,010,254	45
NSW312	fallow	18,805,396	18,511,183	0
NSW319	fallow	32,784,176	32,523,031	6
NSW329	fallow	11,918,979	11,729,223	0
NSW341	fallow	37,909,513	37,488,996	14
NSW346	fallow	32,272,656	32,006,392	0
VIC82	fallow	20,491,907	20,065,307	82893
VIC87	sambar	13,436,391	13,203,248	11
VIC90	sambar	20,627,878	20,396,594	14
VIC93	fallow	24,334,139	23,999,221	23
VIC96	fallow	18,646,001	18,383,346	15
VIC104	sambar	12,409,927	12,235,576	18

PE: paired end

Table S2: Predicted cleavage sites determined by multiple alignments with other *Bopivirus* sequences.

	**Deer**	**Bovine**		**Ovine**	**Caprine**
	**MZ436972**	**KM589358**	**MW298059**	**MW298057**	**MW298058**
**VP4/VP2**	L/A	L/A	L/A	L/A	L/A
**VP2/VP3**	E/G	E/G	E/G	E/G	E/G
**VP3/VP1**	Q/D	Q/D	Q/D	E/G	E/G
**VP1/2A**	E/A	E/D	E/D	E/D	E/D
**2A/2B**	G/P	G/P	G/P	G/P	G/P
**2B/2C**	E/D	E/D	E/D	E/D	E/D
**2C/3A**	E/D	E/D	E/D	E/D	E/D
**3A/3B**	Q/S	E/S	E/S	Q/G	Q/G
**3B/3C**	E/D	E/D	E/D	E/D	E/D
**3C/3D**	Q/L	Q/T	Q/T	E/I	E/T
